# Densely charged polyelectrolyte-stuffed nanochannel arrays for power generation from salinity gradient

**DOI:** 10.1038/srep26416

**Published:** 2016-05-19

**Authors:** Su Hong Kwak, Seung-Ryong Kwon, Seol Baek, Seung-Min Lim, Young-Chang Joo, Taek Dong Chung

**Affiliations:** 1 Department of Chemistry, Seoul National University, Seoul 08826, Korea; 2 Department of Materials Science and Engineering, Seoul National University, Seoul 08826, Korea; 3 Advanced Institutes of Convergence Technology, Suwon-Si, Gyeonggi-do 16229, Korea

## Abstract

We devised anodized aluminium oxide (AAO) frame-supported polyelectrolytic ion-exchange membranes for the application of electrical power generation systems where salinity differences are present. A series of polyelectrolytic AAO membranes (PAMs) were fabricated as a function of concentration of monomers and cross-linkers. Of the ion-selective PAMs as made, the membranes from the most concentrated monomers and cross-linkers, C-PAM100 and A-PAM100, showed the highest area resistances and permselectivities (the resistances were 4.9 and 2.9 Ω · cm^2^, the permseletivities for C-PAM100 and A-PAM100 were 99 and 89%, respectively). The measured resistances and permselectivities allowed the power density to be estimated for C-PAM100 and A-PAM100, 3.5 W/m^2^, and experimentally obtained power density using a reverse electrodialysis (RED) stack was 17.3 mW/m^2^. In addition, we investigated the influence of an AAO framework on a membrane resistance by comparing the PAMs with polyelectrolyte-stuffed capillaries, revealing that the resistance of the PAM has plenty of potential to be further reduced by optimizing the AAO pore spaces.

Electrical power generation from renewable energy resources, e.g. sun, wind, waves, and tidal power, has recently attracted much attention of scientists in opposition to environmental contamination that originates from burning fossil fuels worsen with increasing energy demands. RED is one of the promising processes generating electrical power from renewable energy resources. In the early 1950s, Pattle introduced the concept of electrical power generation exploiting permselective membrane in which cations or anions selectively diffuse from a concentrated solution, e.g. sea water, to a diluted one, e.g. river water, for the first time^1^. There are still critical issues for practical applications, for instance, high cost and short durability of membranes. In particular, biofouling seriously shortens the life time of membranes^2^,^3^. Natural water contains a variety of substances that can cause mechanical damages to the membranes or block the charge transport pathways in the membranes.

Recently, functionalized nanochannel was suggested as a potential alternative to polymer-based ion-selective membranes for energy harvesting devices^4^,^5^. For the purpose of miniaturized power generators and micro batteries, nanofluidic channel may be more suitable structure compared to conventional organic membranes^6^,^7^ because inorganic nanochannels can provide more robust framework^8^. Surface-charged nanochannels counteract their charge imbalance by drawing counter-ions toward its surface while repelling co-ions in the opposite direction, forming electrical double layers (EDLs). Owing to the EDL overlap in nanochannel, counter-ions preferentially pass through the nanochannel from a concentrated solution to a diluted one. Such charge-selective transport of ions generates electrical potential gradient coming from the salinity differences. However, electrostatic functionalization on the inner wall of nanochannel has several limitations in terms of power generation. Manufacturing densely packed nanochannel arrays with linear and evenly distributed pores is an intricate process and only limited number of materials are available for fabrication of nanochannel arrays (e.g. AAO)^9^,^10^,^11^. More importantly, ion selectivity substantially decreases when the nanochannels are situated in a concentrated solution (e.g. sea water) because the EDL could become too thin to get overlapped^12^. To retain high ion-selectivity even in the condition of the concentrated electrolyte solution, surface charge commensurate with the electrolyte concentration is required, but it obviously has upper limit. Another way to do so is to reduce the diameter of channels to tens of nanometers or less^12^,^13^. Nanochannel arrays with tens of nanometer scale is also a challenge to fabricate in terms of technology well as cost.

On the other hand, polyelectrolytes are the polymers that have electrostatic charges on the backbones of those. They are readily hydrated and show low resistivity, ion selectivity, and biocompatible properties when in the form of hydrogel. Harnessing these characteristics, novel *iontronic* devices such as diodes^14^,^15^,^16^, transistors^17^,^18^,^19^, and logic circuits^20^,^21^ were reported recently, suggesting information processor operating in aqueous media. Polyelectrolytes in aqueous solution, however, hardly exist stand-alone maintaining a given shape without framework. This property hinder practical applications of polyelectrolytes for functional devices and ion-selective membranes with wide area. In this sense, we hypothesized that nanochannel array could serve as a framework physically supporting polyelectrolytes and limited ion-selectivity of nanochannel itself could be compensated by polyelectrolytes in it. Specifically, AAO offers rigid framework and polyelectrolyte in its nanochannels does high ion selectivity as well as conductivity. Here we report how polyelectrolyte can be stuffed in the nanochannels of a porous AAO array and how organic-inorganic hybrid materials as made work.

## Results and Discussion

We silanized the AAO surface to functionalize its nanochannel arrays (~200 nm in diameter and 60 μm in length). For the preparation of cation-selective polyelectrolytic AAO membrane (C-PAM), (3-aminopropyl)triethoxysilane (APTES) was used to make the surface positively charged so that 2-acrylamido-2-methyl-1-propanesulfonic acid (AMPSA) monomers should be attracted as counter ions into AAO channels. Similarly, we introduced 3-(trihydroxysilyl)-1-propanesulfonic acid (TPA) to the AAO surface to get dialylldemethyl-ammonium chloride (DADMAC) monomers into the channels for preparation of anion-selective PAM (A-PAM).

**Figure 1:**
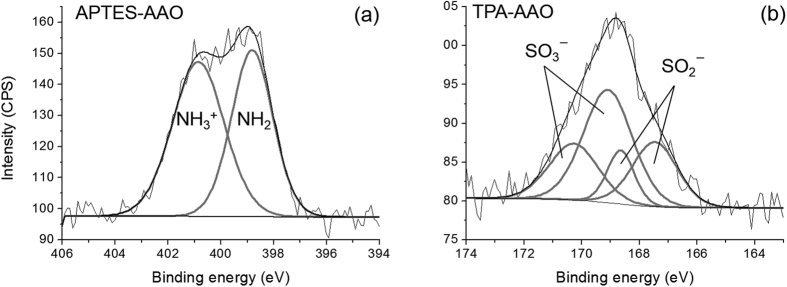
XPS spectra of bare surface of AAO (**a**), APTES silanized AAO surface (**b**) and TPA silanized AAO surface (**c**).

**Figure 2:**
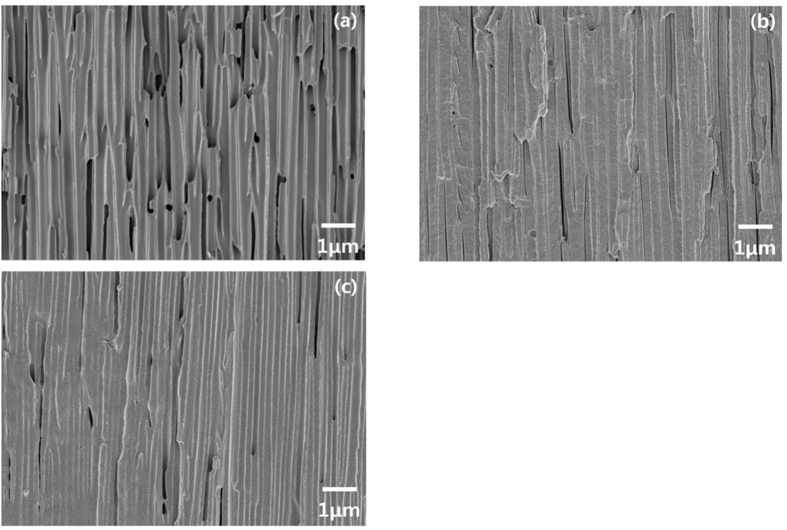
Scanning electron microscopy (SEM) images of cross sectional area of bare AAO (**a**), C-PAM (**b**) and A-PAM (**c**).

**Figure 3:**
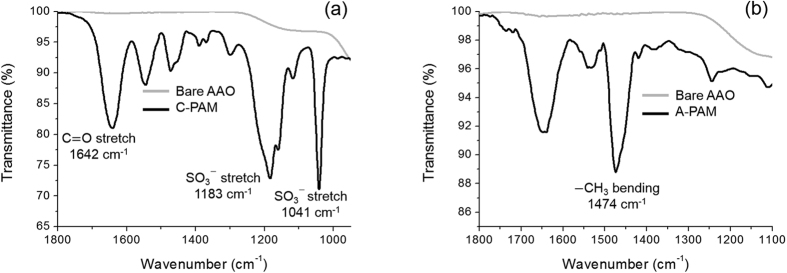
ATR-FTIR spectra of polyDADMAC-filled (**a**) and polyAfilled AAO membranes (**b**). Solid line and gray line indicate polyelectrolyte-filled AAO and bare AAO respectively.

The silanization results on the AAO channel surface were confirmed by X-ray photoelectron spectroscopy (XPS) (Fig. 1). The N1s XPS spectrum of the APTES-modified AAO resulted from overlapping of two peaks; NH_2_ at 398.8 eV and NH_3_^+^ at 400.8 eV (Fig. 1a)^22^,^23^,^24^. In contrast, the bare AAO surface presented a broadened weak peak near 399 eV (Fig. S4). It indicates the successful modification of the surface with APTES as intended. We also verified TPA functionalization on the AAO surface as evidenced by 2 species of SO_2_^−^ (167.5 eV for S2p3/2 and 168.6 eV for S2p1/2) and SO_3_^−^ (169.1 eV for S2p3/2 and 170.3 eV for S2p1/2) from TPA-modified AAO (Fig. 1b and Fig. S5)^25^,^26^. After the polymerization at the AAO support, we probed the polyelectrolytes created in the AAO channel by scanning electron microscopy (SEM) and attenuated total reflectance-Fourier transform infrared (ATR-FTIR) spectra. The cross section of the bare AAO surface showed linear, empty, and uniform pore arrays (Fig. 2a). As a consequence of the polymerization, all AAO arrays are filled with the polyelectrolytes (Fig. 2b,c). We further compared the PAMs with the bare AAO surface as a control by means of ATR-FTIR. The C-PAM exhibited the characteristic peaks of polyAMPSA at 1642, 1183, and 1041 cm^−1^, representing C=O stretching, asymmetric SO_3_^−^, and symmetric SO_3_^−^ stretching vibrations, respectively (Fig. 3a)^27^,^28^. The A-PAM also showed a strong peak at 1474 cm^−1^ which is the fundamental frequency for CH_3_ bending vibrations of polyDADMAC whereas the bare AAO arrays showed no discrete peaks in the spectra (Fig. 3b)^29^. Consequently, the AAO arrays were full of organic polyelectrolytes, yielding organic-inorganic hybrid membranes with linear nanochannels that serve as a mechanical skeleton and minimize the resistance for ion transport.

Next, we evaluated fundamental electrochemical characteristics of the PAMs as ion-exchange membranes. Permselectivity can be calculated from the electrical potential difference that is developed across a membrane:


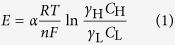


where *E* is the electrical potential of the membrane, *α* is the permselectivity, *n* is the valence, *R* is the gas constant, *T* is the temperature, and *γ* is the mean activity coefficient of NaCl solution (0.686 for 0.51 M and 0.878 for 0.017 M NaCl solution)^30^. *C*_H_ and *C*_L_ represent high and low molar concentrations.

**Figure 4:**
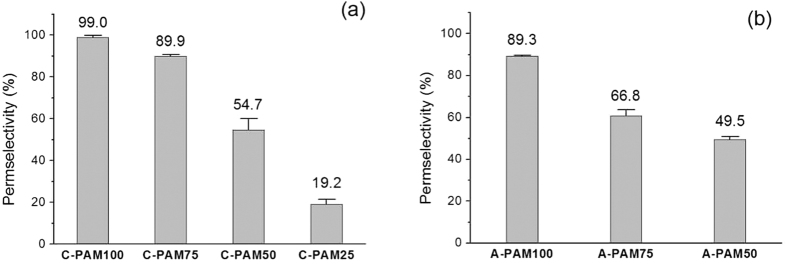
Permselectivities of the C-PAMs (**a**) and the A-PAMs (**b**). The error bars represent the standard deviations obtained from three independent measurements.

We fabricated a series of C-PAMs and A-PAMs varying the concentration of monomers and cross-linkers to investigate the membrane characteristics and optimize the conditions for membrane preparation. The concentration of monomers and cross-linkers increased from C-PAM25 and A-PAM50 to C-PAM100 and A-PAM100 (Table 1). It should be noted that we excluded the irreproducible data of A-PAM25 because the concentrations of the monomer and the cross-linker were too low to be polymerized. Additionally, for the case of A-PAM50, several polymer fragments and unoccupied spaces with polyelectrolytes were observed along the nanochannels, indicating that the concentrations of the monomer and the cross-linker were still insufficient to stuff whole nanochannels with the polyelectrolytes after the polymerization (Fig. S6). As expected, the permselectivity of the PAMs gradually increased in proportion to the monomer concentration (Fig. 4) owing to increasing density of electrostatic charge. When the concentration of monomers and cross-linkers was almost saturated in the solution, C-PAM100 and A-PAM100 reached the highest permselectivities out of the PAMs tested (C-PAM100 99% and A-PAM100 89%). Therefore, we confirmed that the polyelectrolyte-filled AAO membranes provided excellent permselectivities which were not obtainable using only bare AAO membranes (4.4% permselectivity as an AEM based on our experimental data).

Eelectrochemical impedance spectroscopy (EIS) offers the information about the area resistance of the membranes. EIS has been widely used to investigate the electrochemical properties of ion-exchange membranes because it enables to extract individual components from the apparent total impedance, unlike direct current (DC) method^31^. The impedance of a membrane system comes from diffusion boundary layer (DBL), EDL, membrane itself, and solution (Fig. S7). We selectively obtained the resistances of the membrane and solution (R_M+S_) by applying a sinusoidal modulation of 0.3 mA at a frequency of 1 kHz. At such a high frequency, impedances of EDL and the DBL can be assumed to be negligible because of their capacitance in parallel^32^. Since the solution resistance (R_S_) was measured in the absence of the membrane under the same experimental condition, we were able to calculate the pure membrane resistance (R_M_) by subtracting R_S_ from the R_M+S_.

**Figure 5:**
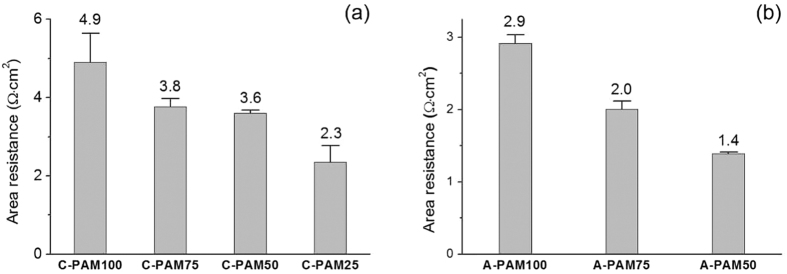
Area resistance of the C-PAMs (**a**) and the A-PAMs (**b**). The error bars indicate the standard deviations of three independent measurements.

Similar to the results of the permselectivity, the area resistance of the membranes was proportional to the concentration of monomers and cross-linkers (Fig. 5). Higher density of the polyelectrolyte leads to better permselectivity due to more electrostatic charge within given volume of the polymer network. However, the ion penetration through the AAO nanochannel is impeded by the densely packed polymer structures. Slower ion penetration means higher membrane resistance, resulting in lower ion current density. The area resistances of the A-PAMs were lower than the C-PAMs (Fig. 5) while the permselectivities of the C-PAMs were higher than the A-PAMs (Fig. 4). There should be a tradeoff between the membrane permselectivity and the area resistance. Therefore, the concentration of the monomers and the cross-linkers should be optimized to obtain the best membrane for maximum electrical power density.

Resistivity of polyelectrolytes in a capillary was measured for the comparison study with that of PAMs (Fig. S8). All conditions for polymerization were the same except the pores in which it occurred. The resistivity of C-PAM100 was about 110-fold higher than that of the polyAMPSA in the capillary (the polyAMPSA in the capillary 7.27 Ω · cm and the C-PAM100 817 Ω · cm). In the case of A-PAM100, the resistivity was about 8-fold higher than that of the polyDADMAC in the capillary (the polyDADMAC in the capillary 69.3 Ω · cm and the A-PAM100 543 Ω · cm). Interestingly, resistivity of the capillary filled with polyelectrolytes, polyDADMAC, was about 9-fold higher than that of polyAMPSA. In contrast, AAO membrane polyDADMAC, A-PAM100, showed about 2-fold lower resistivity than that of polyAfilled AAO membrane, C-PAM100. In order to raise the power, there are a few detailed issues to address. There may be voids unfilled with the polyelectrolytes in the nanochannels where ultra violet light does not reach during the photopolymerization. Minimizing the voids by sophisticatedly adjusting the experimental conditions would lead to reliable performance as well as higher power. Porosity and length of the nanochannels of AAO framework are another parameters that need to be tuned further. Permselectivity and resistivity sensitively reflect the quality of the polyelectrolytes-AAO hybrid membranes so that the experiments shown in this work could provide canonical protocol and setup for optimization of organic charge-selective stuff in inorganic channel framework.

We then estimated theoretical power densities based on the data of the permselectivities and the area resistances that were measured. Recently, P. Długołęcki *et al*. calculated the theoretical power output using a range of properties of commercially available membranes and achieved the fair comparison between the membranes under equal conditions^33^. The theoretical power output was calculated by Equation (2):


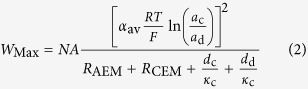


where *N* is the number of membrane pairs, *A* is the area of membrane, α_av_ is the average permselectivity, *R*_AEM_ is the anion exchange membrane resistance, *R*_CEM_ is the cation exchange membrane resistance, *d* is the thickness of solution channel, and *κ* is the conductivity. *a*_c_ and *a*_d_ stand for the activities for the concentrated and diluted solution, respectively. We supposed that *d*_c_ and *d*_d_ are 150 μm and *κ*_d_ and *κ*_c_ are 0.192 and 4.684 S/m, respectively. Equation (2) includes the membrane potential and the resistances of the membranes and the solutions, while excluding necessary pumping energy for water supplies, resistances of concentration polarization, and shadow effects caused by spacers^34^. For a CEM membrane, *α*_av_ is replaced by the CEM permselectivity ignoring the AEM resistance. The solution channel thickness is divided by two.

**Figure 6:**
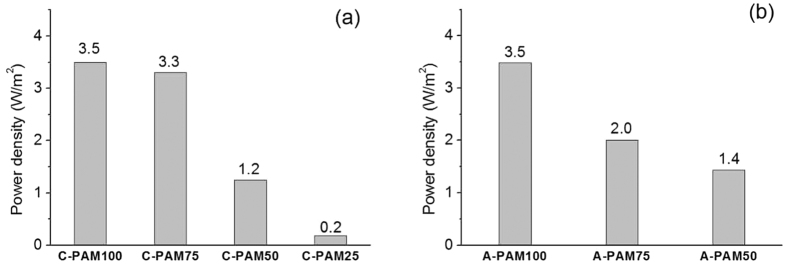
Estimated theoretical power densities of the C-PAMs (**a**) and the A-CAMs (**b**). The theoretical power densities were calculated from the average of the permselectivities and the resistances from Figs 4 and 5, respectively.

Based on the permselectivities and the area resistances recorded, we calculated theoretical power densities of RED systems consisting of the proposed polyelectrolyte-AAO hybrid membranes (Fig. 6). The theoretical power densities of the C-PAM100 and the A-PAM100 were 3.5 W/m^2^, comparable to commercially available membranes^33^. In addition, when compared with bare AAO membranes as AEMs, based on the obtained experimental data, the PAMs showed much higher power density than the bare AAO membrane (0.011 W/m^2^ calculated from 1.34 Ω · cm^2^ area resistance and 4.4% permselectivity).

**Figure 7:**
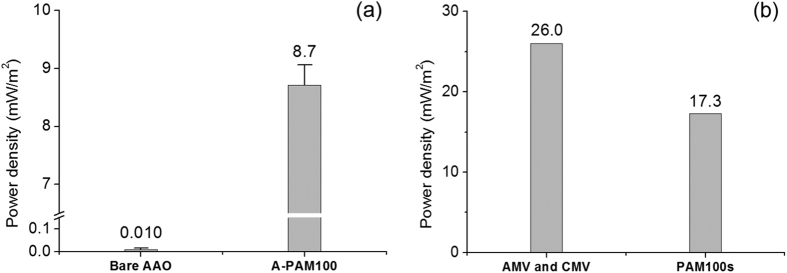
Experimentally measured power densities of a membrane (**a**) and a pair of membranes (**b**). The error bars in (**a**) represent the standard deviations obtained from three independent measurements. The power densities of PAM100s and commercial membranes in (**b**) were measured using an RED stack.

We finally determined maximal power densities experimentally. For a fair comparison, all of the experimental conditions were set to equal except the membrane of interest (Figs S9 and S10). Positively surface-charged AAO membranes as AEMs were compared with A-PAM100s that produced much higher power density than the bare AAO (the A-PAM100 8.7 mW/m^2^ and the bare AAO 0.010 mW/m^2^). These results are much lower than the theoretical values (Fig. 7a). The power produced by PAMs and commercial membranes was also measured using an RED stack specifically designed for the measurement of two membranes^35^. As predicted theoretically, the power density of the PAMs (17.3 mW/m^2^ from a C-PAM100 and an A-PAM100) was comparable to that of the commercial membranes (26.0 mW/m^2^ from a CMV and an AMV) under equal conditions (Fig. 7b). However, the power densities were still much lower compared to the theoretical values and other reported data. J. Veerman *et al*. achieved 1.18 W/m^2^ power density with 5 membrane pairs (CMV and AMV) and 200 μm thick solution channel^36^ and M. C. Hatzell and B. E. Logan obtained ~0.14 W/m^2^ using a single cell pair stack (CMV and AMV) with salinity ratio of 100^35^. Meanwhile, the power density in this study was obtained with a pair of membranes, ~1.3 mm solution channel thickness, and the salinity ratio of 30. A thick solution channel is well known contributor to the reduced power density^37^. In addition, RED stack configuration such as the conduit for solution feed may result in the reduced power density.

## Conclusions

We have successfully developed the organic ion-selective polyelectrolytes supported by the inorganic frameworks of AAO for the application of RED. The PAMs exhibited excellent permselectivities and low area resistances comparable to the commercially available ion-exchange membranes. The density of the polyelectrolytes in the PAMs was found to strongly affect the membrane characteristics that are closely linked to the electrical power production, especially for the RED application. We investigated the effects of the AAO framework on the membrane resistance. The results indicate that the combined system of charge-selective organic material filled in inorganic nanochannel framework has great potential for further advance in functional ion transport. For instance, the tendency found in the data strongly imply that the area resistance of the PAMs could be improved by adjusting the geometric features of AAO arrays, e.g. widening pore spaces or reducing membrane thickness. In spite of its intuitive structural interest inspiring futuristic engineering, intrinsically AAO itself is mechanically brittle, which one of the serious obstacles for practical applications. By integrating with organic polymers, its physical weakness is expected to be relieved, at least maintained, acting as a framework. The ion-selective PAMs in this work suggest a new strategy for portable energy harvesting devices where the concentration gradients present. Miniaturized power supply systems such as micro batteries to operate biosensors for the point-of-care system are another possible targets that may be benefited by the ion-selective PAMs.

## Methods

### Materials

All chemical reagents were purchased from Sigma-Aldrich Inc. if not indicated otherwise. Dialylldemethyl-ammonium chloride (DADMAC, 348279), 2-acrylamido-2-methyl-1-propanesulfonic acid (AMPSA, 282731), (3-aminopropyl)triethoxysilane (APTES, A3648), potassium persulfate (216224), 2-hydroxy-4′-(2-hydroxyethoxy)-2-methylpropiophenone (410896), *N,N*′-methylene-bisacryl-amide (146072) were used without further purification. Anodized aluminum oxide membranes (AAO, No. 6809-6022) were purchased from Whatman Ltd. 3-(trihydroxysilyl)-1-propanesulfonic acid (TPA, SIT8378.3) was from Gelest Inc. Selemion CMV and AMV were purchased from Asashi Glass Co., Ltd.

### Fabrication of ion-selective PAMs

An AAO (200 nm in diameter and 60 μm thickness) was used as a membrane framework. It was dipped in a solution of cationic or anionic monomer, DADMAC or AMPSA. Cation-selective polyelectrolytic AAO membrane (C-PAM) was prepared by the following process. Firstly, an AAO was immersed in dry acetone containing 5% (v/v) APTES for 1 h. After washing with 100 mL acetone, the AAO was placed on a hotplate at 120 °C at 20 min to ensure silanization. Using a syringe pump, an AMPSA monomer solution containing potassium persulfate and N,N′-methylene-bisacrylamide was pushed into the AAO channels. Then the AAO membrane was baked in an oven at 80 °C for 30 min. For preparation of an anion-selective polyelectrolytic AAO membrane (A-PAM), an AAO membrane was reacted with 1 mM TPA in ethanol for 30 min and washed with ethanol. Then the AAO membrane was baked at 70 °C overnight for the complete silanization. The arrays of silanized AAO membrane was filled with a DADMAC solution with 2-hydroxy-4′-(2-hydroxyethoxy)-2-methylpro-piophenone and *N,N*′-methylene-bisacrylamide. Finally, the AAO membrane was exposed to the UV light (365 nm, 16 mW/cm^2^) for 17.5 s for the photopolymerization.

### A series of C-PAMs and A-PAMs

To investigate the density effect of the polyelectrolytes on the membrane properties, the PAMs were synthesized using various concentrations of monomers and cross-linkers (Table 1). The concentration ratio of the monomer to the cross-linker was fixed to maintain chemical structures of polyelectrolytes unchanged.

### Membrane potential measurement

Membrane potential was measured from the potential difference between a pair of Ag/AgCl reference electrodes that were in two compartments divided by the membrane of interest (Fig. S1). A potentiostat (CHI660a, CH Instrument) read an open circuit potential (OCP) from two reference electrodes immersed in 0.017 M and 0.510 M NaCl solutions. The volume of reservoirs was 10 mL and the diameter of membrane was 2 cm. OCP was measured once the membrane potential reached equilibrium.

### Area resistance of a membrane

Area resistance of a membrane was determined by EIS (Fig. S2). Applying sinusoidal modulation of 0.3 mA at a frequency of 1 kHz to the pair of Ag/AgCl planar electrode (1 cm^2^), a galvanostat (Gamry reference600, Gamry instrument) read potential difference between two Ag/AgCl reference electrodes dipped in each compartment filled with 20 mL of 0.5 M NaCl solution. The potential drop across the membrane gave the information about the membrane impedance.

### Resistivity measurement of a polyelectrolyte-stuffed capillary

A capillary filled with polyelectrolytes was 5 cm long and 0.8 mm in diameter. The polymerization process was the same as that for PAM fabrication. Resistivity of polyelectrolytes was measured using EIS (Fig. S3). The experimental parameters of the EIS were the same as for the measurement of the membrane area resistance. The capillary was connected the two reservoirs filled with 0.5 M NaCl solution.

### Power measurement of a membrane

Power density of a membrane was measured with a pair of Ag/AgCl plate electrodes immersed in 0.017 M NaCl solution and 0.51 M NaCl solution (Fig. S9). The distance from the Ag/AgCl electrode to the membrane was 1.3 mm. Chronopotentiometry (CHI660a, CHI Instrument) was used for the power measurements.

### Power measurement of a pair of membranes

We mostly adopted an RED stack configuration developed by M. C. Hatzell and B. E. Logan (Fig. S10)^35^. Briefly, silicon gaskets separating the membranes and create a flow path between the membranes were cut to provide a cross section area (8 cm^2^ for the commercial membranes, CMV and AMV, and 3.14 cm^2^ for the PAMs) and the gasket thickness was ~1.3 mm. Power measurements were conducted based on chronopotentiometry (CHI660a, CH Instrument). Two platinum electrodes at the end of the RED stack introduced current into the system while two Ag/AgCl reference electrodes read out the potential of the membranes. All measurements were carried out without spacers and with 5 mL/min flow rate. The pumping energy for the solution supplies was disregarded in calculating power density.

## Additional Information

**How to cite this article**: Kwak, S. H. *et al*. Densely charged polyelectrolyte-stuffed nanochannel arrays for power generation from salinity gradient. *Sci. Rep.* **6**, 26416; doi: 10.1038/srep26416 (2016).

## Supplementary Material

Supplementary Information

## Figures and Tables

**Table 1:** Recipe for the preparation of C-PAMs and A-PAMs.

	**AMPSA (M)**	**Cross-linker (μM)**	**Initiator (μM)**
C-PAM100	2.81	75.4	43.0
C-PAM75	2.11	56.6	43.0
C-PAM50	1.41	37.7	43.0
C-PAM25	0.70	18.9	43.0
	**DADMAC (M)**	**Cross-linker (μM)**	**Initiator (μM)**
A-PAM100	4.20	67.6	46.5
A-PAM75	3.15	50.7	46.5
A-PAM50	2.10	33.8	46.5

## References

[b1] Pattle R. Production of electric power by mixing fresh and salt water in the hydroelectric pile. Nature 174, 660 (1954).

[b2] Post J. W., Hamelers H. V. M. & Buisman C. J. N. Influence of multivalent ions on power production from mixing salt and fresh water with a reverse electrodialysis system. J. Membrane Sci. 330, 65–72 (2009).

[b3] Vermaas D. A., Kunteng D., Saakes M. & Nijmeijer K. Fouling in reverse electrodialysis under natural conditions. Water Res. 47, 1289–1298 (2013). 23266386 10.1016/j.watres.2012.11.053

[b4] Choi E., Kwon K., Kim D. & Park J. Tunable reverse electrodialysis microplatform with geometrically controlled self-assembled nanoparticle network. Lab Chip 15, 168–178 (2015). 25328008 10.1039/c4lc01031k

[b5] Kim D.-K., Duan C., Chen Y.-F. & Majumdar A. Power generation from concentration gradient by reverse electrodialysis in ion-selective nanochannels. Microfluid. Nanofluid. 9, 1215–1224 (2010).

[b6] Energy conversion in microsystems: is there a role for micro/nanofluidics? Lab Chip 7, 1234–1237 (2007). 17896005 10.1039/b712893m

[b7] Wang W. . Three-dimensional Ni/TiO2 nanowire network for high areal capacity lithium ion microbattery applications. Nano Lett. 12, 655–660 (2012). 22208851 10.1021/nl203434g

[b8] Chu K.-L., Shannon M. A. & Masel R. I. An improved miniature direct formic acid fuel cell based on nanoporous silicon for portable power generation. J. Electrochem. Soc. 153, A1562–A1567 (2006).

[b9] Liu N.-W. . Fabrication of anodic-alumina films with custom-designed arrays of nanochannels. Adv. Mater. 17, 222–225 (2005).

[b10] Asoh H. . Fabrication of ideally ordered anodic porous alumina with 63 nm hole periodicity using sulfuric acid. J. Vac. Sci. Technol. B 19, 569–572 (2001).

[b11] Masuda H. . Highly ordered nanochannel-array architecture in anodic alumina. Appl. Phys. Lett. 71, 2770–2772 (1997).

[b12] Boo H. . Ionic strength-controlled virtual area of mesoporous platinum electrode. J. Am. Chem. Soc. 126, 4524–4525 (2004). 15070363 10.1021/ja0398316

[b13] Cheng L.-J. & Guo L. J. Rectified ion transport through concentration gradient in homogeneous silica nanochannels. Nano Lett. 7, 3165–3171 (2007). 17894519 10.1021/nl071770c

[b14] Han J. H. . Ion flow crossing over a polyelectrolyte diode on a microfluidic chip. Small 7, 2629–2639 (2011). 21780313 10.1002/smll.201100827

[b15] Fang J. . Conjugated zwitterionic polyelectrolyte as the charge injection layer for high-performance polymer light-emitting diodes. J. Am. Chem. Soc. 133, 683–685 (2010). 21171591 10.1021/ja108541z

[b16] Cayre O. J., Chang S. T. & Velev O. D. Polyelectrolyte diode: nonlinear current response of a junction between aqueous ionic gels. J. Am. Chem. Soc. 129, 10801–10806 (2007). 17691778 10.1021/ja072449z

[b17] Seo J. H. . Improved injection in n-type organic transistors with conjugated polyelectrolytes. J. Am. Chem. Soc. 131, 18220–18221 (2009). 19968301 10.1021/ja908441c

[b18] Herlogsson L. . Downscaling of organic field‐effect transistors with a polyelectrolyte gate insulator. Adv. Mater. 20, 4708–4713 (2008).

[b19] Lee S. W. . Periodic array of polyelectrolyte-gated organic transistors from electrospun poly (3-hexylthiophene) nanofibers. Nano Lett. 10, 347–351 (2009). 19994870 10.1021/nl903722z

[b20] Han J. H., Kim K. B., Kim H. C. & Chung T. D. Ionic circuits based on polyelectrolyte diodes on a microchip. Angew. Chem. Int. Ed. 48, 3830–3833 (2009). 10.1002/anie.200900045 19378316

[b21] Chun H. G. & Chung T. D. Iontronics. Annu. Rev. Anal. Chem. 8, 441–462 (2015). 10.1146/annurev-anchem-071114-040202 26048549

[b22] Acres R. G. . Molecular structure of 3-aminopropyltriethoxysilane layers formed on silanol-terminated silicon surfaces. J. Phys. Chem. C 116, 6289–6297 (2012).

[b23] Jani A. M. M. . Pore spanning lipid bilayers on silanised nanoporous alumina membranes. Proc. of SPIE: Melbourne, Australia. 7267, 0T01–0T10, doi: 10.1117/12.808769 (2008, December).

[b24] Martin H. J., Schulz K. H., Bumgardner J. D. & Walters K. B. XPS study on the use of 3-aminopropyltriethoxysilane to bond chitosan to a titanium surface. Langmuir 23, 6645–6651 (2007). 17488131 10.1021/la063284v

[b25] Nasef M. M. & Saidi H. Surface studies of radiation grafted sulfonic acid membranes: XPS and SEM analysis. Appl. Surf. Sci. 252, 3073–3084 (2006).

[b26] Nasef M. M., Saidi H., Nor H. M. & Yarmo M. A. XPS studies of radiation grafted PTFE‐g‐polystyrene sulfonic acid membranes. J. Appl. Polym. Sci. 76, 336–349 (2000).

[b27] Xu G., Gao S., Ji X. & Zhang X. Characterization and Synthesis Mechanism of Nanosilver/PAMPS Composites by Microwave. Soft Nanoscience Letters 4, 15–23 (2014).

[b28] Rastogi P. K., Krishnamoorthi S. & Ganesan V. Synthesis, characterization, and ion exchange voltammetry study on 2‐acrylamido‐2‐methylpropane sulphonic acid and N‐(hydroxymethyl) acrylamide‐based copolymer. J. Appl. Polym. Sci. 123, 929–935 (2012).

[b29] Bhalerao U. M., Acharya J., Halve A. K. & Kaushik M. P. Controlled drug delivery of antileishmanial chalcones from Layer-by-Layer (LbL) self assembled PSS/PDADMAC thin films. RSC Adv. 4, 4970–4977 (2014).

[b30] Dlugolecki P. E. Mass transport in reverse electrodialysis for sustainable energy generation. (University of Twente, 2009). 10.1021/es9009635 19764265

[b31] Park J. S., Choi J. H., Woo J. J. & Moon S. H. An electrical impedance spectroscopic (EIS) study on transport characteristics of ion-exchange membrane systems. J. Colloid Interf. Sci. 300, 655–662 (2006). 10.1016/j.jcis.2006.04.040 16730020

[b32] Długołecki P. . On the resistances of membrane, diffusion boundary layer and double layer in ion exchange membrane transport. J. Membrane Sci. 349, 369–379 (2010).

[b33] Dlugolecki P., Nymeijer K., Metz S. & Wessling M. Current status of ion exchange membranes for power generation from salinity gradients. J. Membrane Sci. 319, 214–222 (2008).

[b34] Długołȩcki P., Gambier A., Nijmeijer K. & Wessling M. Practical potential of reverse electrodialysis as process for sustainable energy generation. Environ. Sci. Technol. 43, 6888–6894 (2009). 19764265 10.1021/es9009635

[b35] Hatzell M. C. & Logan B. E. Evaluation of flow fields on bubble removal and system performance in an ammonium bicarbonate reverse electrodialysis stack. J. Membrane Sci. 446, 449–455 (2013).

[b36] Veerman J., De Jong R., Saakes M., Metz S. & Harmsen G. Reverse electrodialysis: Comparison of six commercial membrane pairs on the thermodynamic efficiency and power density. J. Membrane Sci. 343, 7–15 (2009).

[b37] Vermaas D. A., Saakes M. & Nijmeijer K. Doubled power density from salinity gradients at reduced intermembrane distance. Environ. Sci. Technol. 45, 7089–7095 (2011). 21736348 10.1021/es2012758

