# Supplementary material for: Densely charged polyelectrolyte-stuffed nanochannel arrays for power generation from salinity gradient
Source: Sci Rep. 2016 May 19;6:26416. doi: 10.1038/srep26416 (PMC4872233; doi:10.1038/srep26416)
Supplement: Supplementary Information [file srep26416-s1.pdf]

Supporting Information for

Densely charged polyelectrolyte-stuffed nanochannel arrays for power generation from salinity gradient

Su Hong Kwak, Seung-Ryong Kwon, Seol Baek, Seung Min Lim, Young-Chang Joo, and Taek Dong Chung

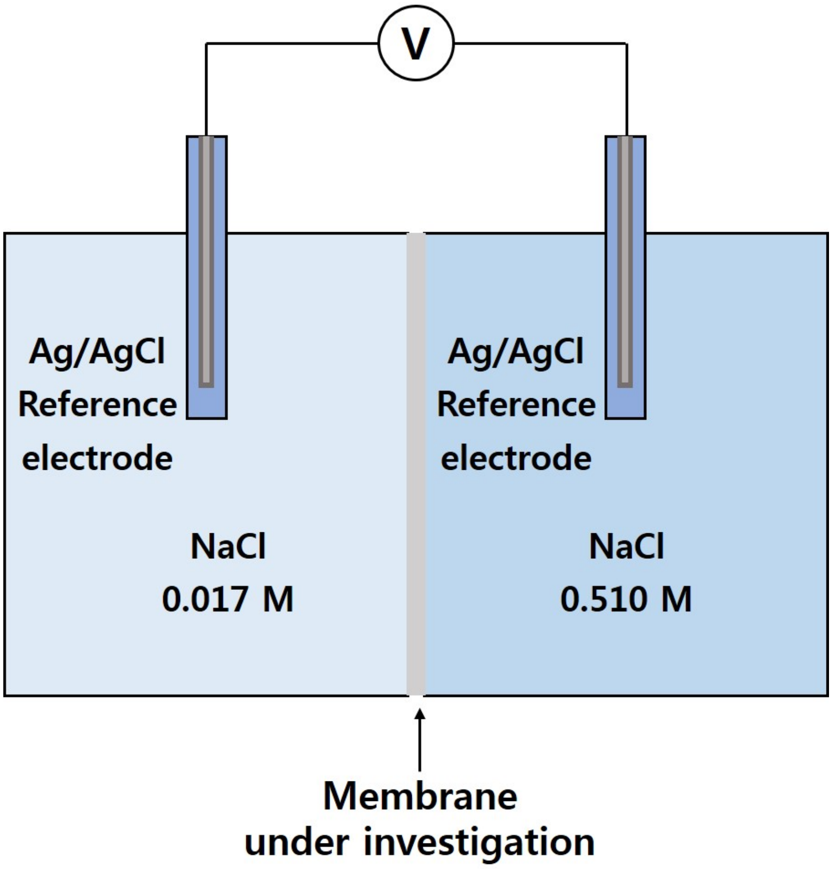

Figure S1. Scheme of experimental setup to measure permselectivity of ion exchange membrane.

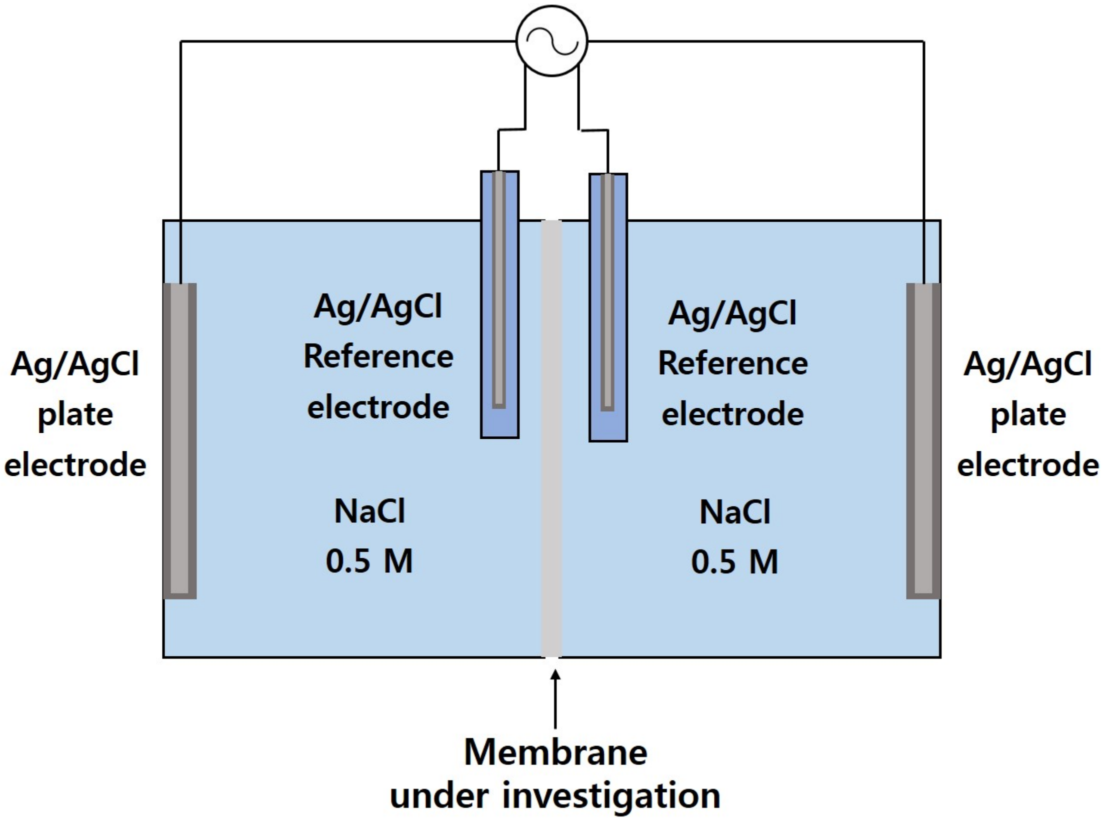

Figure S2. Scheme of experimental setup to measure the area resistance of ion exchange membrane.

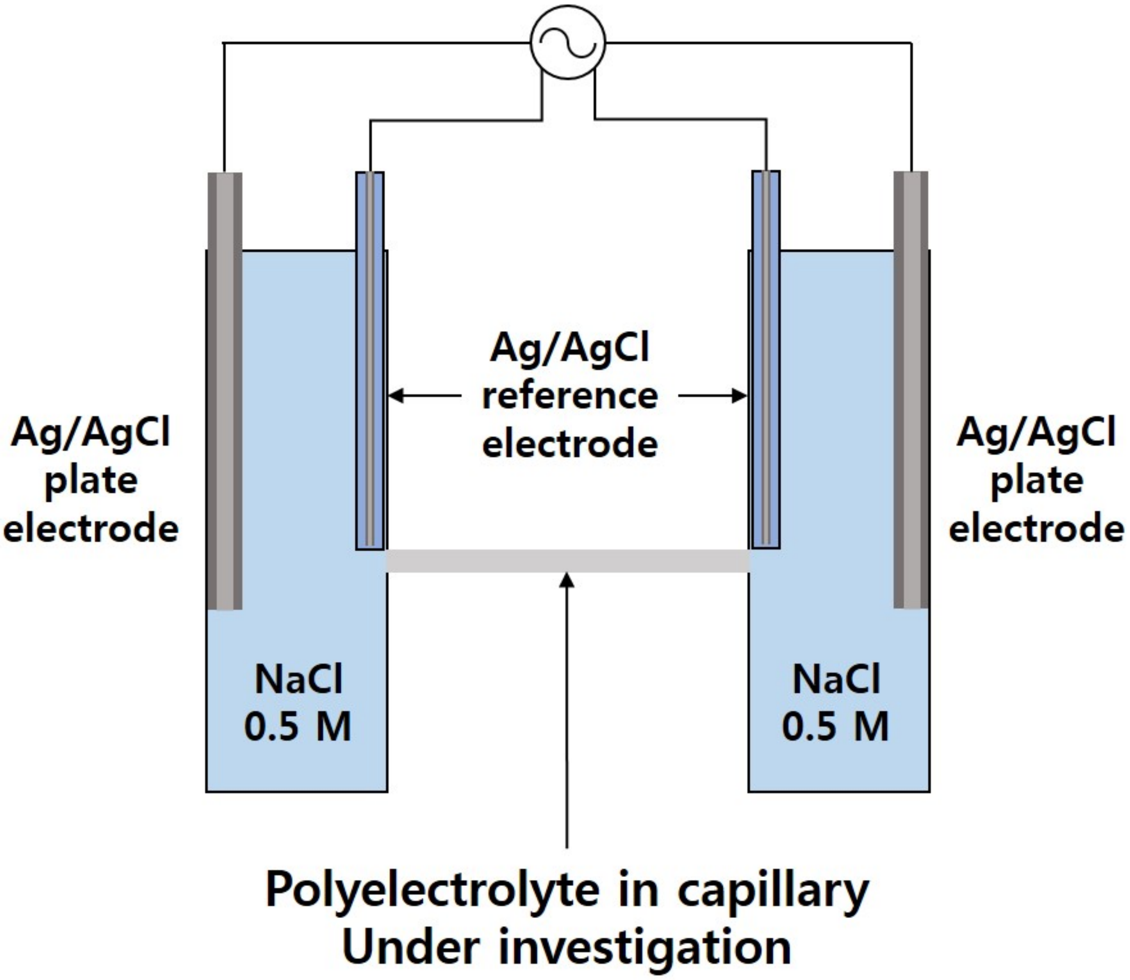

Figure S3. Scheme of experimental setup to measure the resistivity of polyelectrolyte in capillary. The capillary is 5 cm long and the cross section area of the polyelectrolyte phase interfaced with the solution is 0.8 mm in diameter.

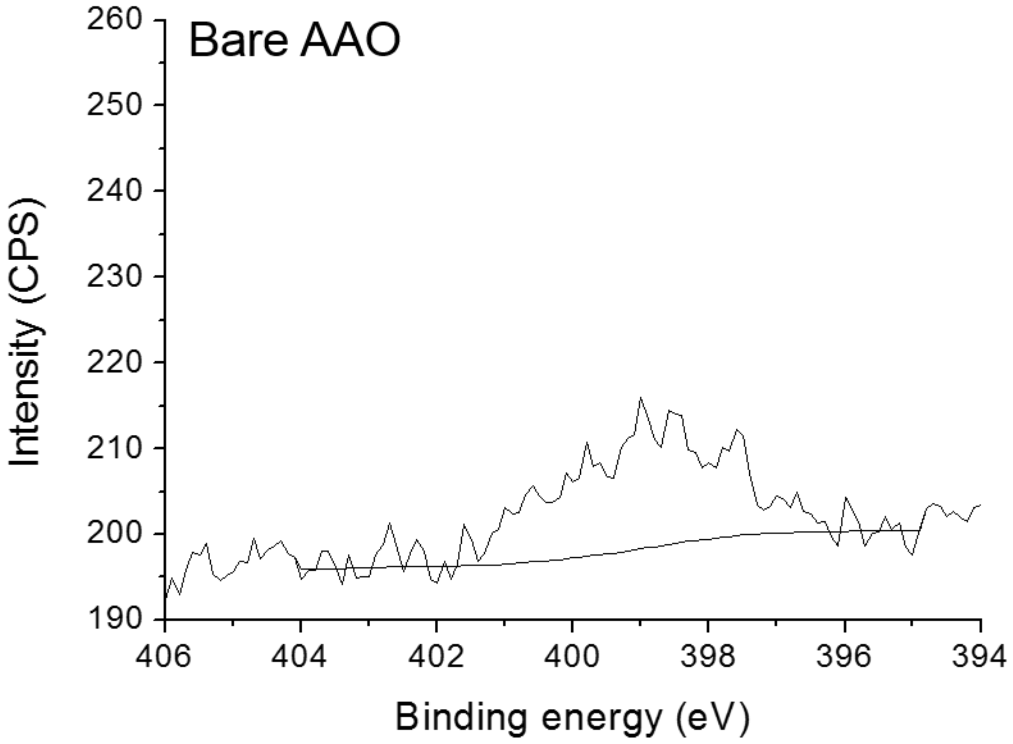

Figure S4. XPS Spectrum of N1s peak on the bare surface of AAO.

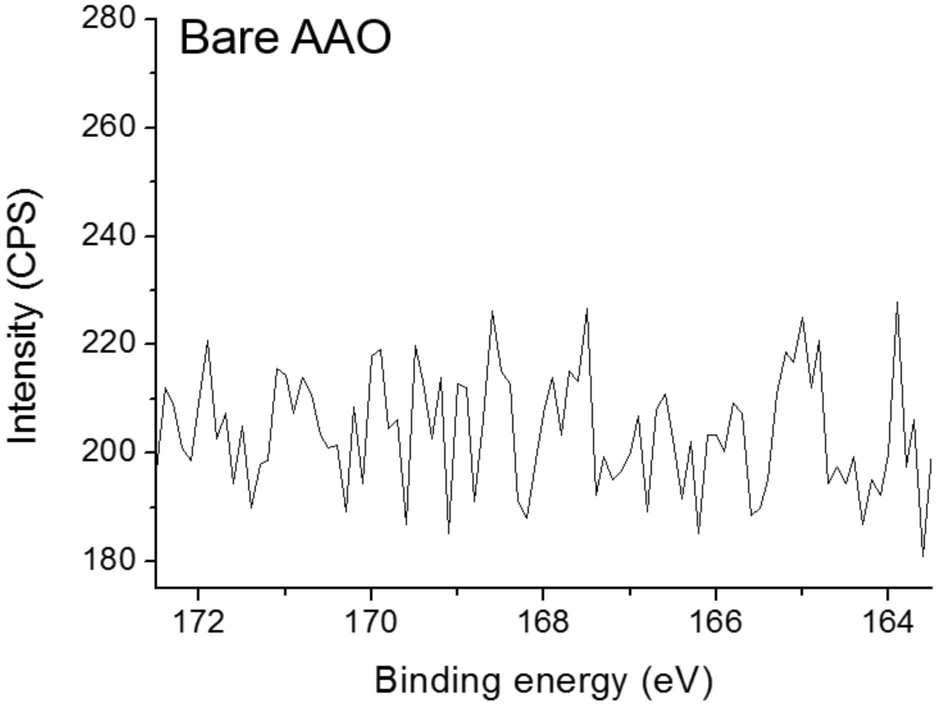

Figure S5. XPS Spectrum of S2p on the bare surface of AAO.

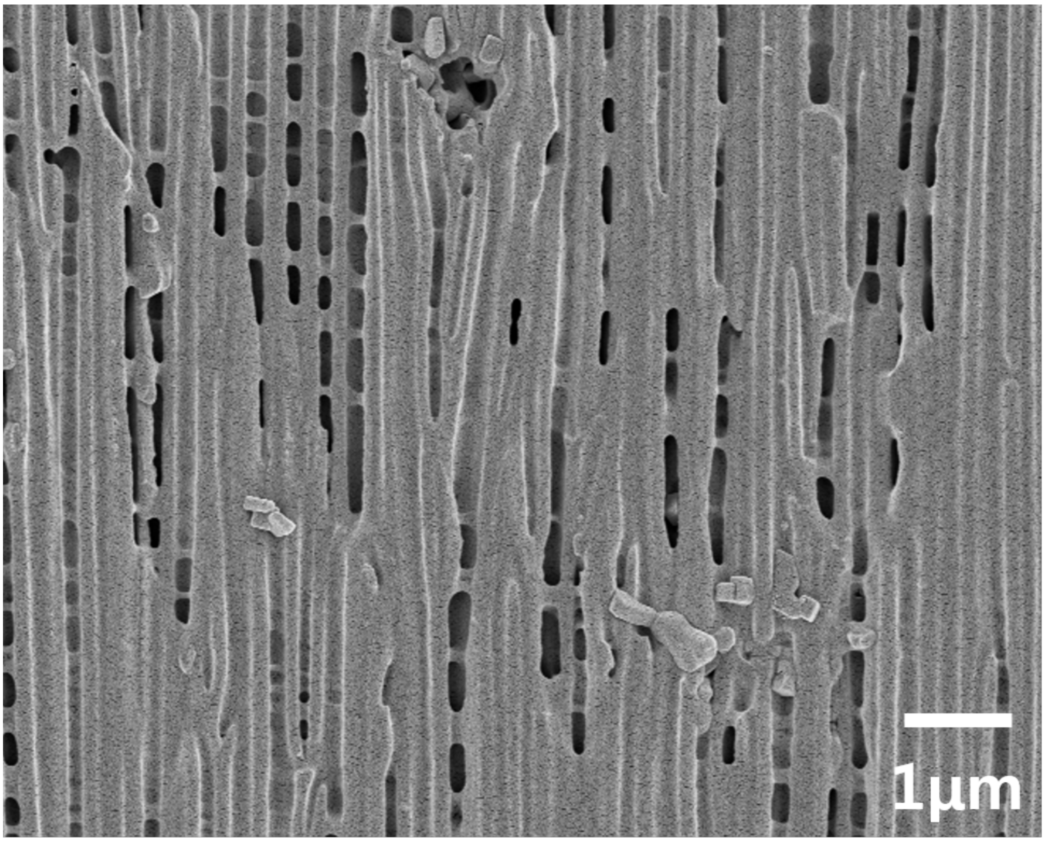

Figure S6. An image of scanning electron microscopy showing a cross sectional area of A-PAM50.

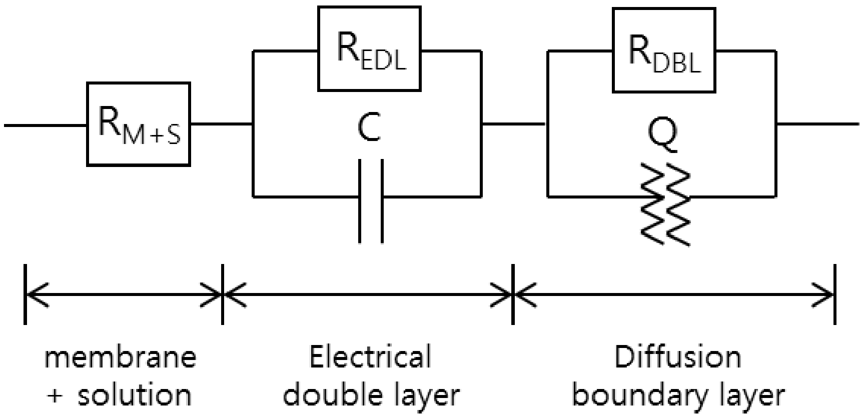

Figure S7. Total impedance of ion-exchange membrane. R_{M+S} is the sum of membrane and solution resistance and R_{EDL} is the resistance of the counter-ion transfers from the solution to the membrane through the electrical double layer (EDL). C is the capacitance of the ionic charge at the EDL and R_{DBL} is the resistance of diffusion boundary layer. Q is the constant phase element representing non-ideal capacitance of the DBL.

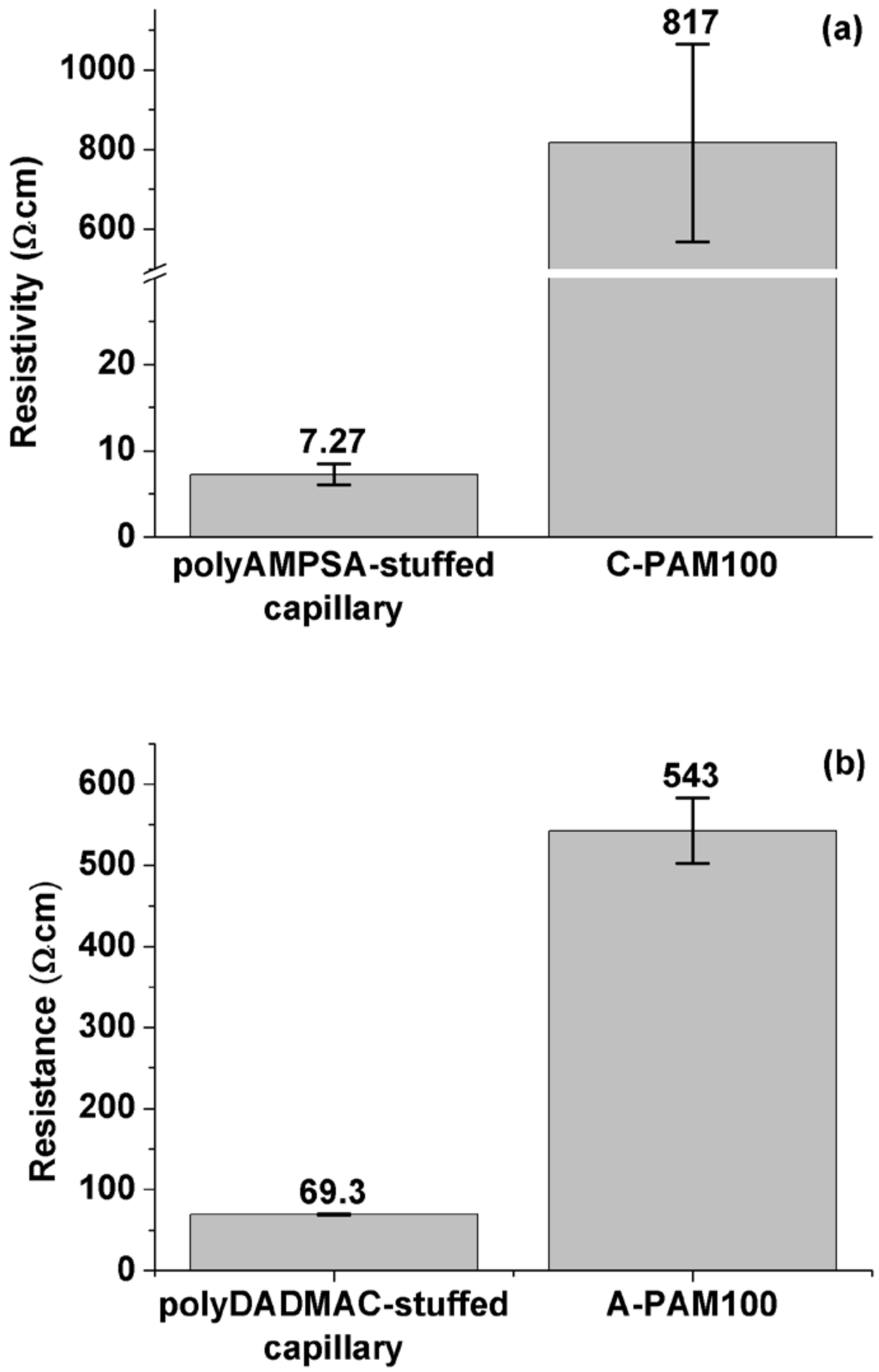

Figure S8. Resistivity comparison between the polyelectrolyte-stuffed capillary and the PAM100s; (a) C-PAM100 and (b) A-PAM100.

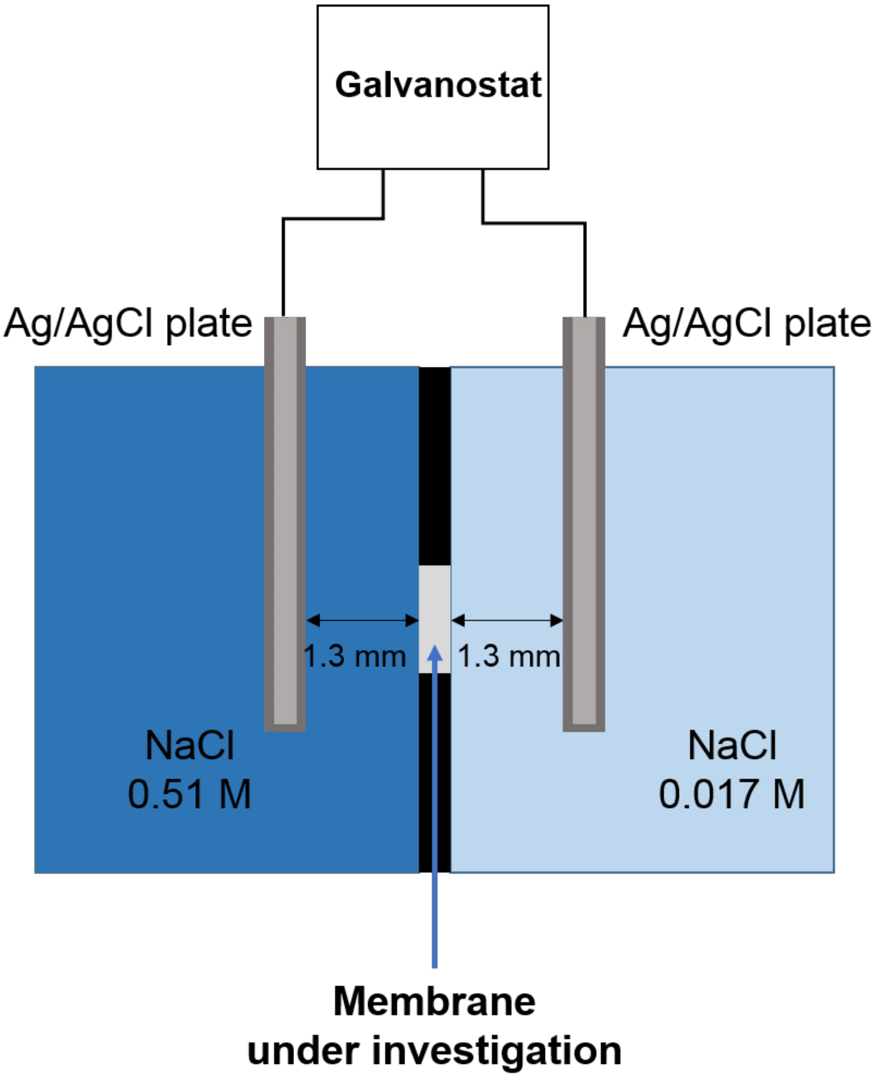

Figure S9. Scheme of experimental setup to measure maximal power density of a membrane.

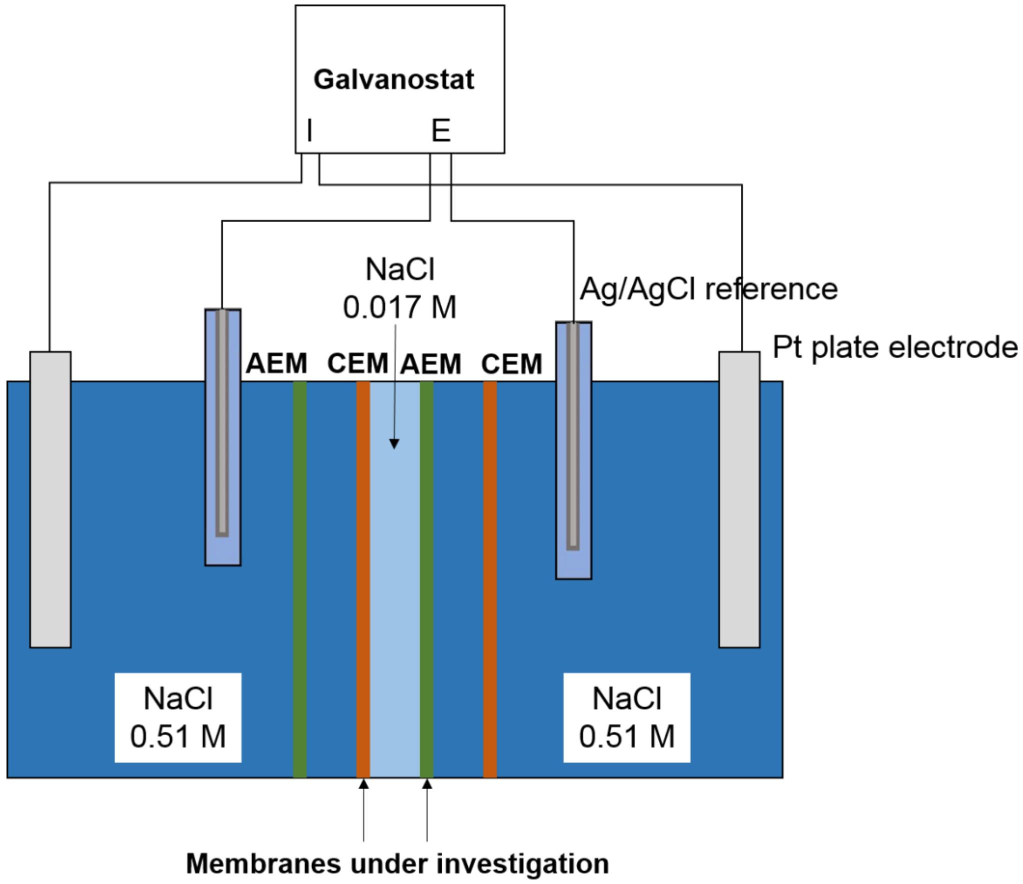

Figure S10. Scheme of experimental setup to measure maximal power density of a membrane pair. Flow rate for solution supplies was 5 mL/min.
